# Supplementary material for: Complexome profiling of the Chlamydomonas psb28 mutant reveals TEF5 as an early PSII assembly factor
Source: Plant Cell. 2025 Mar 17;37(6):koaf055. doi: 10.1093/plcell/koaf055 (PMC12164590; doi:10.1093/plcell/koaf055)
Supplement: koaf055_Supplementary_Data [file koaf055_supplementary_data.zip › psb28_interactive.zip!/plots/intensity/qualityPlots/peptides/PFH7_Cre14.g626200.t1.1.html]

### Cre14.g626200.t1.1

Trivial name: PFH7  
SeqCoverage [median]: 14.80  
Mass: 30.85 kDa  
  
 

| Sequence | MissedCleavage | Mass [Da] | wtIntensity | muIntensity | Score |
| --- | --- | --- | --- | --- | --- |
| IVVLDPDAR | 0 | 996.56 | 266.24 | 1362.74 | 73.26 |
| LGSATDRTGTGSHEL | 1 | 1500.72 | 327.51 | 0.00 | 48.11 |
| SGVVDTGSGGSVVSDIR | 0 | 1590.78 | 329.30 | 2176.22 | 166.60 |
|  |  |  | 923.0 | 3539.0 |  |
